# Exploiting Post-mitotic Yeast Cultures to Model Neurodegeneration

**DOI:** 10.3389/fnmol.2018.00400

**Published:** 2018-11-02

**Authors:** Andrea Ruetenik, Antonio Barrientos

**Affiliations:** ^1^Department of Neurology, School of Medicine, University of Miami Miller School of Medicine, Miami, FL, United States; ^2^Neuroscience Graduate Program, School of Medicine, University of Miami Miller School of Medicine, Miami, FL, United States; ^3^Department of Biochemistry, School of Medicine, University of Miami Miller School of Medicine, Miami, FL, United States

**Keywords:** inducible yeast model, *Saccharomyces cerevisiae*, chronological life span, neurodegenerative disorder, mitochondria, proteotoxicity, reactive oxygen species

## Abstract

Over the last few decades, the budding yeast *Saccharomyces cerevisiae* has been extensively used as a valuable organism to explore mechanisms of aging and human age-associated neurodegenerative disorders. Yeast models can be used to study loss of function of disease-related conserved genes and to investigate gain of function activities, frequently proteotoxicity, exerted by non-conserved human mutant proteins responsible for neurodegeneration. Most published models of proteotoxicity have used rapidly dividing cells and suffer from a high level of protein expression resulting in acute growth arrest or cell death. This contrasts with the slow development of neurodegenerative proteotoxicity during aging and the characteristic post-mitotic state of the affected cell type, the neuron. Here, we will review the efforts to create and characterize yeast models of neurodegeneration using the chronological life span model of aging, and the specific information they can provide regarding the chronology of physiological events leading to neurotoxic proteotoxicity-induced cell death and the identification of new pathways involved.

## Neurodegenerative Diseases Modeled in Yeast

The unicellular yeast *Saccharomyces cerevisiae*, known as baker’s yeast or brewer’s yeast, has been extensively used in the areas of biotechnology and biomedicine. Over the last century, *S. cerevisiae* has been used as a valuable organism for studying the principles of microbiology, characterizing biochemical pathways and understanding the biology of more complex eukaryotic organisms (Botstein, 1991). A multiplicity of basic cellular activities are conserved from yeast to humans, including DNA replication, recombination and repair, RNA transcription and translation, intracellular trafficking, enzymatic activities of general metabolism and mitochondrial biogenesis, protein quality control pathways, nutrient sensing, and stress resistance pathways (reviewed in Barrientos, 2003). Therefore, knowledge gained in yeast has been fundamental to understanding the physiology of human cells and the pathophysiology of human diseases.

Over the last two decades, yeast has been used to model the human aging process and complex neurodegenerative disorders, including amyotrophic lateral sclerosis (ALS), Parkinson’s disease (PD), and Huntington’s disease (HD) (reviewed in Miller-Fleming et al., 2008). In humans, these neurodegenerative disorders are characterized by the progressive, selective loss of neurons in different areas of the brain associated with the misfolding of disease-specific proteins. Although yeast cells are less complex than human neurons, basic metabolic pathways involved in neurodegeneration are well-conserved in *S. cerevisiae*, as mentioned earlier.

**FIGURE 1:**
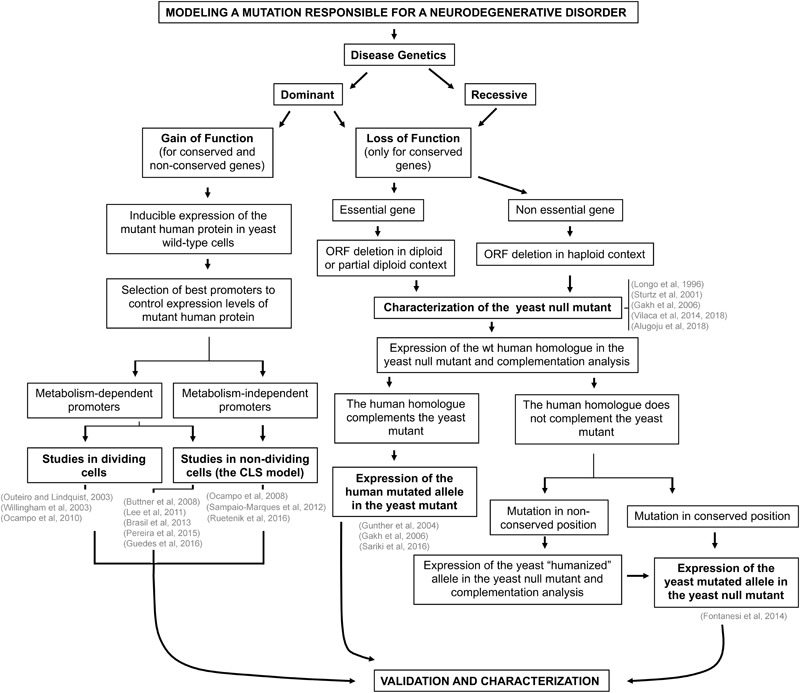
Scheme depicting the strategic planning for the creation of yeast models of neurodegenerative disorders. The strategies used for the construction of yeast models of human monogenic neurodegenerative diseases depend on genetic and pathophysiological constraints. Whether the disease is dominant or recessive, whether the phenotype results from a gain or a loss of function of the protein involved, and whether the gene is functionally conserved or not from yeast to humans are determinants of the kind of yeast model that can be generated. References located in the relevant text boxes provide actual examples in the literature of yeast models of neurodegenerative disorders. See full explanation in the text. Figure modified from Fontanesi et al. (2009).

Constructing a yeast model of a human neurodegenerative disorder does not present major technical difficulties *per se* but requires a carefully designed multistep plan (Figure 1). A major goal is that the yeast model of a particular disease must recapitulate the crucial events preceding cell death that are manifested during the course of the human disorder.

The strategies that are usually followed in the construction of yeast models of human neurodegenerative diseases depend on genetic and pathophysiological constraints. In some cases, human disorders result from a loss of function of the disease gene encoded protein. In these cases, when the human disease gene is conserved from yeast to humans, functional complementation studies will allow determining whether the human disease gene product partially or fully replaces the function of the yeast gene product. If complementation occurs, human disease gene mutant alleles are expressed in yeast and tested for functionality as for mutations in the Cu–Zn superoxide dismutase gene responsible for ALS (Gunther et al., 2004). If complementation does not occur, the disease mutations, frequently involving conserved protein residues, are alternatively introduced in the yeast protein and subsequently analyzed as it has been reported for mutations in the adenine nucleotide translocator (*ANT1*) responsible for cases of external progressive ophthalmoplegia (Fontanesi et al., 2004).

In age-associated neurodegenerative disorders such as Alzheimer’s disease (AD), PD, or HD, the human disease genes are restricted to vertebrates. In these diseases, however, a gain of function of the disease mutant proteins greatly contributed to pathogenicity. Mutant forms of the proteins huntingtin (htt) and α-synuclein, responsible for HD and some familiar forms of PD, respectively, undergo misfolding and damage several cellular structures thus leading to cell death. Yeast models of these disorders are constructed by expressing the human disease gene in yeast thus providing paradigms where the toxic effect of the misfolded protein on the cellular physiology and metabolism can be conveniently studied (Outeiro and Lindquist, 2003; Willingham et al., 2003; Ocampo et al., 2010).

## The Yeast Chronological Life Span Assay: a Better System to Model Neurodegeneration?

### Yeast Aging

The ideal yeast models of age-associated neurodegeneration should incorporate the concept of cellular aging. Yeast has two life spans; a replicative life span (RLS), defined as the number of daughters produced by each dividing mother cell, and a chronological life span (CLS), defined as the capacity of stationary (Go) cultures to maintain viability over time. The CLS assay, initially established by Valter Longo (University of Southern California) (Longo et al., 1996, 2012; Fabrizio et al., 2001; Fabrizio and Longo, 2007), has been proposed to reflect aging in post-mitotic mammalian cells, such as neurons (Chen et al., 2005; Braun et al., 2009). Despite the different level of complexity between yeast and human post-mitotic cells, yeast models of aging have been instrumental for the identification of essential conserved pathways that influence healthspan and life span. For example, studies aiming to understand the molecular mechanisms of calorie-restriction (CR)-mediated longevity, allowed for the identification of several longevity genes (reviewed in Ruetenik et al., 2016). In yeast, CR down-regulates the conserved Ras/cAMP/PKA, TOR, and Sch9 signaling pathways that integrate the nutrient and other environmental cues to regulate cell growth, division and life span (Wei et al., 2008). Deletion of *RAS2, TOR1*, or *SCH9* enhances cellular protection against thermal and oxidative stresses and extends yeast CLS (Longo and Finch, 2003). Inhibition of these pathways converges on the activation of stress resistance transcription factors that will induce the expression of cell protection systems (e.g., catalase and superoxide dismutase -SOD2) and accumulation of stored nutrients (trehalose and glycogen). The key components of these pathways also regulate stress resistance and life span in higher eukaryotes (Fontana et al., 2010). For example, both Akt and S6K, homologs of yeast *SCH9*, regulate life span in higher eukaryotes and inhibition of Tor/S6K signaling extends life span in worms, flies, and mice (Paradis et al., 1999; Hertweck et al., 2004; Kapahi et al., 2004; Selman et al., 2009). Also, mice deficient in elements of the Ras pathway have extended health and life span (Yan et al., 2007; Enns et al., 2009). The longevity pathways play an essential role in the regulation of mitochondrial biogenesis and function, crucial for the management of neuronal life and death, in yeast and higher eukaryotes including mammals (Bonawitz et al., 2007; Anderson and Prolla, 2009). For example, deletion of the *TOR1* gene extends yeast CLS in part by increasing mitochondrial mass and respiration (Bonawitz et al., 2007) and by promoting adaptive mitochondrial reactive oxygen species (ROS) signaling (Pan et al., 2011). The Ras/cAMP/PKA pathway senses excessive ROS to signal to the Hap2,3,4,5 transcriptional system and down-regulate mitochondrial biogenesis (Dejean et al., 2002; Chevtzoff et al., 2009). Also in mammals, modulation of mitochondrial biogenesis and metabolism through the Tor, Akt1, and Ras pathways involves the transcriptional co-activator PGC-1α (Anderson and Prolla, 2009). PGC-1α transcriptional activity appears to be induced in the oxidative stress response and CR through a shared mechanism, suggesting that in mammals, regulation of mitochondrial function is a critical element in both cell survival and longevity (Anderson et al., 2008).

Therefore, the yeast CLS model of aging is expected to be an informative paradigm regarding connections between aging and neurodegeneration, at least for those diseases originating from proteotoxic stress.

### The CLS Assay

**FIGURE 2:**
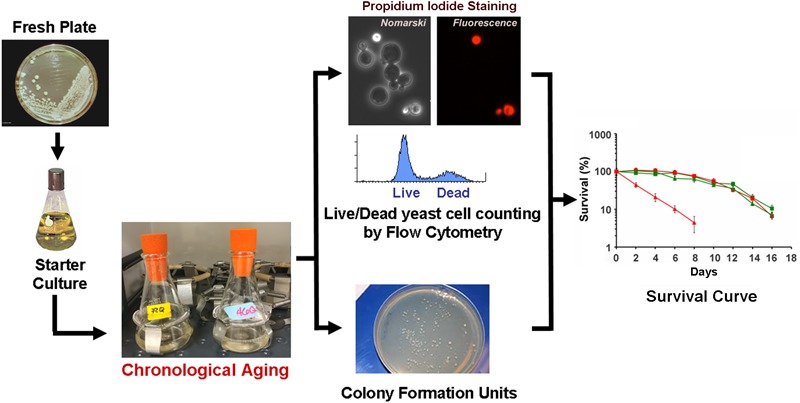
The chronological life span (CLS) assay. In a typical CLS assay, yeast strains from frozen stocks (–80°C) are patched onto YPD agar plates (2% glucose) and incubated at 30°C. The following day, cells are inoculated into 10 ml of SDC media and grown overnight. After 24 h, cells are inoculated into 50 ml of synthetic (SDC) media in 250-ml flasks to an optical density at 600 nm (OD_600_) of 0.250. Cultures are then grown with shaking (250 rpm) at 30°C. We recommend that all flasks are capped using Bio-Silico plugs that ensure sterility and maximize airflow (Hirschmann, Louisville, KY, United States). Maximum cell density is normally reached after 48 h of growth in SDC, therefore we consider 3 days after inoculation as Day 0 of CLS. Subsequently, cellular viability is determined every other day by either a clonogenic approach using the colony formation unit (CFU) assay of propidium iodide staining and flow cytometry analyses (PI-FCM) as described (Ocampo and Barrientos, 2012; Ocampo et al., 2012). The data obtained from viability analysis is then used to construct survival curves.

**FIGURE 3:**
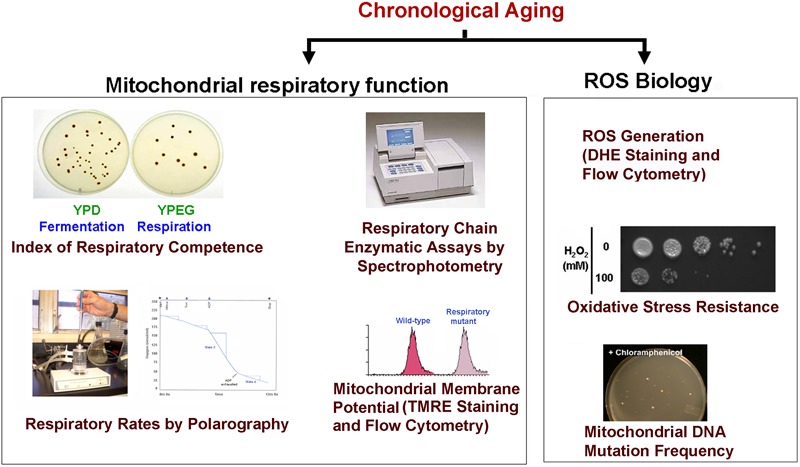
Scheme of some of the experiments used to monitor mitochondrial damage during CLS. During CLS of wild-type yeast cultures expressing or not the toxic proteins are collected at different time points and analyzed for markers of mitochondrial function including: (1) The index of respiratory competence (Parrella and Longo, 2008) or proportion of cells that are able to form colonies in media containing respiratory (glycerol) versus fermentable (glucose) carbon sources. (2) Endogenous cell respiration measured polarographically and mitochondrial respiratory chain (MRC) enzymatic activities measured spectrophotometrically (Barrientos et al., 2009). (3) Mitochondrial membrane potential estimated *in vivo* by flow cytometry using the fluorescent probe tetramethylrhodamine ethyl ester (TMRE) (Ocampo et al., 2010). (4) Oxidative stress and damage: mitochondrial and cellular reactive oxygen species (ROS) production can be measured in whole cells by using the oxidant-sensitive fluorescent probes, MitoSOX Red and dihydroethidium (DHE) as described (Ocampo et al., 2012). Additionally, resistance to oxidative stress can be determined by exposing cells to high H_2_O_2_ concentrations (up to 100 mM) for 1 h prior to testing growth in complete solid media. Also, mtDNA point mutation frequency can be estimated by counting the proportion of cells that acquire resistance to specific antibiotics (chloramphenicol or erythromycin). Cells are plated in respiratory media supplemented with the drugs as described (O’Rourke et al., 2002).

Chronological life span determination must be conducted in exact conditions to ensure reproducibility. CLS is classically determined in cells grown in liquid synthetic complete media containing 2% glucose (SDC) supplemented with standard amounts of amino acids and nucleotide bases as previously described (Sherman, 1991; Ocampo and Barrientos, 2011). The assay is depicted in Figure 2 and briefly explained in the figure legend. During CLS, multiple approaches can be implemented to analyze the progression or decline of physiological features over time, how they are influenced by the expression of toxic proteins, and how they correlate with survival. As an example, an array of assays that can be implemented to analyze mitochondrial function and ROS production is presented in Figure 3.

### Strain Background

When preparing yeast models of aging and neurodegeneration it is important to consider the strain background. Several laboratory strains commonly used in aging research are genetically and physiologically heterogeneous (Mortimer and Johnston, 1986; van Dijken et al., 2000), which is reflected in some manner in their CLS (Fabrizio and Longo, 2007; Ocampo and Barrientos, 2011; Ocampo et al., 2012). For example, two different yeast genetic backgrounds commonly used in this field are the short-lived strains BY4741 (Powers et al., 2006; Murakami et al., 2008; Burtner et al., 2009) and DBY2006 (Bonawitz et al., 2007; Pan and Shadel, 2009). Many commonly used strains, including BY4741, CEN.PK113-7D and DBY2006, are S288c derivatives that carry a mutation affecting Hap1, a heme-dependent regulator of a number of genes involved in electron-transfer reactions (Gaisne et al., 1999). This contributes to their reduced ability to respire compared with strains carrying a wild-type *HAP1* gene such as W303, although other genetic differences between the strains may also contribute (Ocampo et al., 2012). As a consequence, when the cells are grown in synthetic medium containing 2% glucose, the CLS of BY4741 (∼8 days), CEN.PK113-7D (∼9 days), and DBY2006 (∼3 days) is shorter than of W303 yeast (∼10.5 days), and the effect of mutations affecting nutrient-sensing and stress-resistance pathways are not exactly equal (for a comparison of CLS in different yeast strains, see, Ocampo and Barrientos, 2011; Ocampo et al., 2012; Chen and Petranovic, 2015). Therefore, the use of at least two different strain backgrounds is always advised to obtain interpretable results.

### External Modulators of Yeast CLS

Various external conditions have been found to shorten or extend yeast CLS in wild-type cells. Those need to be taken into account when designing the CLS assays with yeast models of neurodegenerative diseases and when interpreting the results obtained. For example, the CLS on the type of medium in which cells are initially grown. Yeast grown in synthetic medium containing dextrose survive for a few days (from 6 to 11 depending on the yeast strain), which is a very short, high-metabolic post-diauxic phase (Sinclair et al., 1998; Longo, 1999). In contrast, wild-type yeast grown in YPD, and maintained on expired YPD medium, can survive for several weeks, and thus have a prolonged period of hypometabolism in stationary phase (Sinclair et al., 1998; Longo, 1999 and reviewed in Chen et al., 2005). Yeast cultures even have slightly longer survival if they are pre-grown in YPEG (respiratory media) (MacLean et al., 2001). Additional modulators of CLS include: pH of the growth culture medium (Burtner et al., 2009; Longo et al., 2012; Ocampo et al., 2012), amino acid content (Johnson and Johnson, 2014; Maruyama et al., 2016), nitrogen source (Hess et al., 2006; Santos et al., 2015), osmolarity (Kaeberlein et al., 2002; Smith et al., 2007; Murakami et al., 2008), and temperature (Smith et al., 2007). All these factors need to be carefully controlled and maintained constant through experimentation since they are significant sources of experimental variability.

## CLS Studies Using Yeast Models of Neurodegenerative Disease

Although, the CLS assay has been used extensively to study and identify modifiers of normal yeast aging, proportionately few studies have employed the CLS assay to study yeast models of neurodegenerative disease. This represents a vast missed opportunity. A summary of studies conducted to date using the CLS assay to evaluate the toxicity of disease-related proteins in a wild-type yeast background as well as studies that attempted to find toxicity suppressors in these models using the CLS assay are listed in Table 1. In this section, we will briefly introduce the main findings of these studies, the study limitations, and our advice for future work. As seen in Table 1, the majority of these studies also quantified yeast viability throughout the CLS assay using only the colony formation unit (CFU) assay method, which, as explained previously may affect the strength of the interpretations that can be made from these studies if not combined with the parallel use of the PI–FCM method.

**Table 1:** Yeast models of neurodegenerative disorders established in the context of the chronological life span model of aging.

Disorder	Protein involved	Inducible expression system	Toxic?	Select assays reported	Strain and growth phases studied	CLS evaluation method	Interventions tested for CLS	Reference
Huntington’s disease	Huntingtin	β-Estradiol inducible 103Q-htt expression *TEF1-7* promoter	Yes	103Q expression by fluorescence microscopy Cell respiration Serial dilution growth test Oxidative phosphorylation inhibitors	W303 Exponential Stationary	CFU Live/dead staining	Glucose restriction *HAP4* overexpression Growth in respiratory media	Ruetenik et al., 2016
Alzheimer’s disease	Amyloid-β	Constitutive expression of Amyloid-β with ER targeting signal under multiple promoter types	Yes	CLS (constant pH, oxygen) Serial dilution growth test ROS levels Amyloid oligomerization by immunoblot and immunostaining Proteome activity assay Mitochondrial function Transcriptional and lipid composition response to amyloid-β expression Oxygen limitation Glycogen/trehalose levels	CEN.PK113-7D Exponential Diauxic shift Stationary	CFU Serial dilution Live/dead staining	Glucose restriction	Chen and Petranovic, 2015; Chen et al., 2017
	Mutant ubiquitin (UBB^+1^)	Constitutive expression of UBB^+1^ under *TEF1* promoter in single or multi-copy vectors	No	Proteolytic activity assay Induced protein misfolding challenge ROS levels by DHR123 staining TUNEL assay Caspase activation Heat shock/oxidative stress resistance Transcript levels by rtPCR Knockout of *Atg1*	CEN.PK 113-11C Exponential Stationary	CFU Serial dilution Live/dead staining	N/A	Munoz-Arellano et al., 2018
Parkinson’s disease	α-Synuclein	Galactose inducible expression of wild-type and A53T mutant α-synuclein at different intensities	Yes	ROS levels by DHE staining Annexin V/PI/TUNEL staining	BY4741 Stationary	CFU Live/dead staining	*aif1, yca1, nma111* deletion Depletion of mtDNA	Buttner et al., 2008
		Constitutive expression of α-synuclein under *TPI* promoter	Yes	Autophagy activity assay Autophagic activity by western blotting	BY4741 Stationary	CFU	Glucose restriction *tor1* deletion	Guedes et al., 2016
		Doxycycline induction at different times during CLS, Galactose induction at stationary phase Constitutive expression of wild-type and mutant α-synuclein	Yes	ROS levels by DHE staining Autophagy/mitophagy induction by mRNA levels, activity assay, and confocal microscopy Sod1/2 activity Mitochondrial function	BY4741 and W303 Exponential Diauxic shift Stationary	CFU Live/dead staining	*atg11* and *atg32* deletion Chloroquine supplementation	Sampaio-Marques et al., 2012
		Chromosomally integrated α-synuclein under *FAA2* promoter	Yes	ROS levels by DCFH staining Oxidative stress resistance	BY4741 Stationary	CFU	Triclabendazole or Albendazole supplementation	Lee et al., 2011
	Synphilin-1	Constitutively expressed wild-type and mutant synphilin-1 under *TPI1* promoter	Yes	Protein processing by immunoblotting Aggregate formation by fluorescent microscopy Serial dilution growth test ROS levels by DHE staining Annexin V/PI staining	BY4741 Exponential Stationary	CFU	*sir2* deletion	Buttner et al., 2010
	DJ-1	Single deletion of multiple yeast DJ-1 family members	Yes	Autophagic activity by GFP-Atg8 reporter and fluorescent microscopy Heat shock resistance Carbon starvation Rapamycin treatment Gene expression changes upon DJ-1 knockout	BY4742 and BY4743 Exponential Stationary	CFU	N/A	Miller-Fleming et al., 2014
	Parkin	Constitutive expression of Parkin under *GPD* promoter	No	Oxidative stress resistance Parkin localization by immunoblotting Pink1 overexpression Autophagy disruption Parkin localization and aggregation by fluorescent microscopy	W303 Stationary	CFU	Growth in respiratory media	Pereira et al., 2015
Congenital neuronal ceroid lipofuscinosis, Alzheimer’s disease risk, others	Pep4	*pep4* deletion strain	Yes	Serial dilution growth test Stress resistance tests ROS levels by H_2_DCFDA staining Mitochondrial morphology	BY4741 Exponential Stationary	CFU Live/dead staining	Quercetin supplementation	Alugoju et al., 2018
Familial amyotrophic lateral sclerosis (ALS)	SOD1	Wild-type and AV4 mutant human *SOD1* under yeast *SOD1* promoter	No	SOD1 activity assay Intracellular oxidation analysis Protein carbonylation assay	BY4741 Exponential Stationary	CFU	*gsh1* deletion	Brasil et al., 2013
		*SOD1* and *SOD2* deletion strains	Yes	Oxygen consumption, amino acid requirements	DBY746 and W303 Exponential Diauxic shift Stationary	CFU	Low aeration *coq3 deletion*	Longo et al., 1996
		*SOD1* deletion strains	Yes	Mitochondrial fractionation for protein localization SOD enzymatic activity	DBY746 and W303 Exponential Stationary	Serial dilution	*CCS1* overexpression	Sturtz et al., 2001
Fronto-temporal lobar degeneratio/n (FTLD-U), ALS	TDP-43	Galactose inducible expression of wild-type or mutant *TDP-43*	Yes	ROS levels by DHE staining Aggregation formation by fluorescent microscopy Annexin V/PI staining Respiratory capacity	BY4741 Exponential Stationary	CFU	Depletion of mtDNA Deletion/inhibition of respiratory complexes	Braun et al., 2011
Ataxia with oculomotor apraxia type 2 (AOA2) Amyotrophic lateral sclerosis 4 (ALS4)	Sen1	Multiple genetically modified mutant *sen1* strains	Yes	Serial dilution growth test Mitochondrial function by fluorescent microscopy and flow cytometry ROS levels by DHE and H_2_DCFDA staining Stress resistance Transcriptome analysis of Sen1 mutants Annexin V/PI staining	BY4741 Exponential Stationary	CFU	N/A	Sariki et al., 2016
Friedreich ataxia	Frataxin (Yfh1p)	Wild-type or mutant *YFH1* expressed into *yfh1* deletion strain under endogenous *YFH1* promoter	Yes	Serial dilution growth test Oxiblot Iron challenge ROS damage Growth in low oxygen	BY4741 Exponential Stationary	CFU	N/A	Gakh et al., 2006
Niemann–Pick type C	NPC1	*ncr1* deleted yeast cells	Yes	Sphingolipid analysis B-Galactosidase activity Oxidative stress resistance Mitochondrial function Mitochondrial network by fluorescent microscopy Serial dilution growth test	BY4741 Exponential Post-diauxic shift Stationary	CFU	Deletion of *SIT4, CDC55, PKH1*, or *SCH9* Treatment with myriocin	Vilaca et al., 2014, 2018

*CFU, colony formation unit; CLS, chronological life span.*

### Huntington’s Disease (HD) and Other Polyglutamine (PolyQ) Disorders

Polyglutamine (PolyQ) diseases are caused by a CAG codon repeat expansion in disease-specific genes resulting in the expression of misfolding/aggregation-prone proteins with expanded polyQ stretches. These include HD, characterized by intranuclear and cytoplasmic htt inclusions, and six types of spinocerebellar ataxias (Shao and Diamond, 2007). Mitochondrial dysfunction, altered mitochondrial integrity and dynamics and impaired axonal trafficking have been associated with the pathogenesis of polyQ diseases in human patients and several research model organisms (Panov et al., 2002; Trushina et al., 2004; Cui et al., 2006; Lin and Beal, 2006; Wang et al., 2009). Mutant htt may also damage neurons directly by inducing mitochondrial depolarization and altering calcium homeostasis in patients and in mouse models (Panov et al., 2002). Additionally, mutant htt has been shown to alter mitochondrial function indirectly by inhibiting the expression of the transcriptional co-activator PGC-1α, which regulates mitochondrial biogenesis and respiration (Cui et al., 2006).

#### Yeast Models in Dividing Cells

In yeast, expression of htt exon I fragments comprising the polyQ stretches faithfully recapitulates htt misfolding/aggregation in a polyQ length-dependent manner, as shown by the pioneering work of Dr. Susan Lindquist (Krobitsch and Lindquist, 2000). These models were constructed by placing polyQ expression under the control of a galactose-inducible promoter. Cells were transferred from glucose (or raffinose) to galactose-containing media to induce strong polyQ expression and acute cytotoxicity. Upon polyQ toxicity, several pathways, including ER stress, cytoskeletal disturbances, oxidative stress, and mitochondrial dysfunction were found to contribute to growth arrest and cell death (Meriin et al., 2002; Muchowski et al., 2002; Solans et al., 2006; Duennwald and Lindquist, 2008; Ocampo et al., 2010). These models have been used extensively to search for suppressors of polyQ toxicity. Toxicity can be reduced by enhancing the clearance pathways for the removal of cytoplasmic aggregate-prone proteins by increasing autophagy (Sarkar et al., 2009), activating the ubiquitin–proteasome system (Bauer and Nukina, 2009) or repairing the endoplasmic reticulum-associated degradation (ERAD) pathway (Duennwald and Lindquist, 2008). Toxicity is ameliorated by modulating the chaperone systems involved in protein refolding, aggregation and disaggregation, thus shifting the balance toward the non-toxic species (Krobitsch and Lindquist, 2000; Vacher et al., 2005). Suppression of polyQ toxicity is also achieved by protecting the cells against events downstream from polyQ misfolding and oligomerization, including suppression of cytoskeletal instability (Kaminosono et al., 2008), suppression of the kynurenine pathway to limit the accumulation of toxic metabolites that increase ROS generation (Giorgini et al., 2005) and protection of mitochondrial integrity and function (Ocampo et al., 2010). These yeast models have been also used for genome-wide screens to identify genes that enhance polyQ toxicity. The identified genes clustered in the functionally related cellular processes of response to stress, protein folding, and ubiquitin-dependent protein catabolism (Willingham et al., 2003).

#### Yeast Models in Non-dividing Cells

**FIGURE 4:**
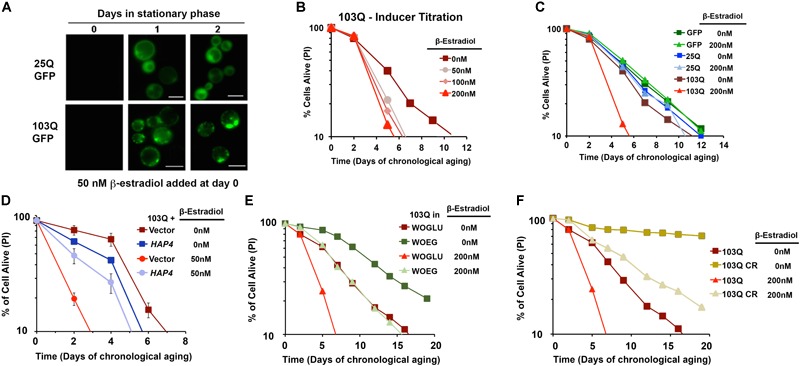
β-Estradiol inducible yeast models of polyglutamine disorders. **(A)** Chronology of polyQ-GFP protein accumulation, followed by fluorescence microscopy, in cells induced with 50 nM β-estradiol. The bar is 5 μm. **(B,C)** Yeast CLS. Survival of wild-type cells expressing 25Q or 103Q from a β-estradiol-inducible promoter activated with the indicated amounts of inducer or supplemented with the solvent (ethanol) was estimated by propidium iodide (PI) staining and flow cytometry analysis of 10,000 cells. Data are average of three samples in % of cells alive at day 0. In **(C)** a β-estradiol titration was performed. **(D)** Effect of increased mitochondrial biogenesis by *HAP4* overexpression on CLS of yeast expressing 103Q from day 0 in the stationary phase. Error bars represent SEM for three independent experiments. **(E)** Effect of growth in synthetic medium containing ethanol and glycerol as non-fermentable (respiratory) carbon sources (WOEG) on 103Q yeast CLS compared to synthetic medium containing glucose as fermentable carbon source (WOGLU). SD <1 for all samples, *n* = 3. **(F)** Effect of calorie restriction (CR) modeled by growing the cells in the presence of 0.5% glucose vs. non-CR (2% glucose) in 103Q yeast CLS. SD <1 for all samples, *n* = 3. This figure was constructed using panels previously published in Ruetenik et al. (2016) with permission since they were published under the terms of the Creative Commons Attribution (CC BY) license.

To ascertain how polyQ toxicity modulates aging, we have studied suppressors of mutant htt toxicity using the CLS assay in a yeast model of HD by expressing 103Q starting at day 0 of the stationary phase using an inducible β-estradiol expression system (Figure 4) (Ruetenik et al., 2016). In the CLS assay, in standard synthetic media growth conditions, we observed that overexpression of this 103Q htt fragment through induction with 50 nM β-estradiol resulted in a severe shortening of CLS compared to control cells (Ruetenik et al., 2016) (Figure 4).

In previous studies with this yeast model, using exponentially-growing cells, we had discovered that the 103Q mutant htt fragment associates with the mitochondrial membrane and disrupts several key mitochondrial functions (Solans et al., 2004; Ocampo et al., 2010). In an independent study, aiming to understand the determinants of CLS in wild-type yeast, we reported that there are mitochondrial respiratory thresholds during the exponential phase of growth, below which yeast CLS is shortened (Ocampo et al., 2012). We were therefore interested in assessing whether increasing the mitochondrial respiratory capacity in our inducible yeast model of HD from culture initiation could partially or entirely suppress the toxicity of 103Q overexpression in the CLS assay. To test this hypothesis, we explored three separate interventions that had previously been shown to increase mitochondrial respiration during exponential growth and to increase overall CLS. The first intervention, was the constitutive overexpression of *HAP4*, the catalytic subunit of the master transcriptional regulator complex that controls the expression of nuclear genes coding for mitochondrial proteins, which we had previously shown increases mitochondrial respiration in dividing cells when overexpressed (Ocampo et al., 2010). We next tested two nutritional modulators of yeast CLS that increase mitochondrial respiration: growth in media containing non-fermentable carbon sources, and glucose restriction. As expected, each of these three interventions did increase mitochondrial respiration exponential growth phase compared to cells, and these interventions did not decrease 103Q expression compared to controls. Importantly, all three of these interventions tested robustly extended the CLS of yeast overexpressing 103Q beyond that of cells overexpressing 103Q without the interventions (Ruetenik et al., 2016) (Figure 4). Therefore, the results in our HD yeast model suggest that treatments targeting mitochondrial biogenesis, and specifically mitochondrial respiration, may be beneficial in the treatment of HD patients to delay neuronal death.

### Alzheimer’s Disease (AD)

Chen and Petranovic (2015) employed the CLS assay to study yeast models of AD and how overexpression of amyloid β peptides affects yeast CLS. In these experiments, the authors studied two different amyloid precursor protein cleavage products, Aβ40 and Aβ42. Importantly, Aβ42 has been found to be more prone to aggregation in previous studies by other groups, and it has been hypothesized that mutations that lead to an increase in Aβ42 production may be one of the primary causes of familial AD (Hampel et al., 2010). The experiments by Chen and Petranovic (2015) explored the effects of the constitutive overexpression, at various levels, of either Aβ40 or Aβ42, both with targeting signals directing the peptides to the endoplasmic reticulum (ER) secretory pathway. CLS studies were conducted at a constant pH of 5.0 and dissolved oxygen levels were kept above 30%, all other conditions being standard. In these conditions, overexpression of ER-targeted Aβ40 or Aβ42 both shortened CLS compared to cells without Aβ expression, with Aβ42 overexpression causing the most robust CLS shortening. Propidium iodide live/dead staining further confirmed these findings, revealing that overexpression of the ER-targeted Aβ42 peptide significantly increased the proportion of dead cells throughout the stationary phase compared to control cells (Chen and Petranovic, 2015). In a second publication by this group, a glucose restriction from the standard 2% concentration to 1% glucose at culture initiation was found to robustly increase CLS in the same model system, measured through propidium iodide staining (Chen et al., 2017).

Munoz-Arellano et al. (2018) have also recently studied the consequences of the expression of the loss-of-function ubiquitin variant UBB^+1^ in yeast using the CLS model. This UBB^+1^ variant, found to be neurotoxic in high levels, specifically accumulate and co-aggregate with the tangles and plaques of AD, as well as in HD and some other diseases of protein misfolding, while, in non-diseased cells the UBB^+1^ variant is degraded albeit inefficiently. UBB^+1^ has a high affinity for the proteasome and thus can make it inefficient to degrading other ubiquitinated moieties. To examine the effects of UBB^+1^ on aging cell health, Munoz-Arellano et al. (2018) expressed UBB^+1^ both at low and high levels. Whereas high expression of UBB^+1^ had no effect on yeast CLS, low UBB^+1^ levels greatly extended CLS, through a mechanism that included reduction of ROS levels and attenuation of apoptosis markers compared to control and high UBB^+1^ expressing cells. Altering the ubiquitin–proteasome system capacity by low UBB^+1^ expression was also found to protect cells against the adverse effects of protein misfolding induced through amino acid analogs (Munoz-Arellano et al., 2018). Further exploitation of this UBB^+1^ yeast model might help to identify additional factors that together with UBB^+1^ may contribute to the neurotoxic proteinotaphy disease initiation and/or progression.

### Parkinson’s Disease (PD) and Other α-Synucleopathies

α-Synuclein (α-syn) is a presynaptic brain protein. Mainly cytosolic, α-synuclein can bind membranes and participate in vesicle trafficking. Misfolded/aggregated α-synuclein is the major constituent of cytoplasmic inclusions called Lewy bodies, a pathological hallmark of α-synucleinopathies (Uversky, 2008). This group of diseases includes PD, multiple system atrophy, and dementia. Mutant α-synuclein, as well as increased α-synuclein levels due to gene duplication, can cause PD. OXPHOS dysfunction and excessive ROS generation have been linked to PD (Schapira et al., 1990; Bender et al., 2006; Kraytsberg et al., 2006; Henchcliffe and Beal, 2008).

#### Yeast Models in Dividing Cells

Yeast models of α-synucleinopathies consist of overexpression of mutant or wild-type forms of α-synuclein (Outeiro and Lindquist, 2003). α-Synuclein misfolding-aggregation cause growth arrest and induces toxicity and cell death in an expression level-dependent manner (Outeiro and Lindquist, 2003). α-Synuclein toxicity in yeast is characterized by altered lipid metabolism, disrupted vesicular trafficking, ER stress, oxidative stress, impaired protein degradation, and mitochondrion-dependent cell death (Outeiro and Lindquist, 2003; Willingham et al., 2003; Kritzer et al., 2009; Yeger-Lotem et al., 2009; Auluck et al., 2010; Donmez et al., 2012). These yeast models have been also used for genome-wide screens to identify genes that enhance α-synuclein toxicity. Different from what it was found in yeast models of polyQ toxicity, the identified genes clustered in the processes of lipid metabolism and vesicle-mediated transport (Willingham et al., 2003).

#### Yeast Models in Non-dividing Cells

Whereas studies in growing cells have provided valuable mechanistic and physiological insight into α-synuclein toxicity, they need to be complemented with studies in non-dividing yeast cells to explore how α-synuclein toxicity modulates aging and *vice versa*. Of the neurodegenerative diseases, PD has been the most extensively studied using yeast models and the CLS assay. Of these studies, four have explored the effects of α-synuclein overexpression using different expression systems. The earliest of these studies, by Buttner et al. (2008), used a galactose-inducible system in which wild-type α-synuclein or α-synuclein with the toxic A53T point mutation, a cause of hereditary PD, was highly expressed in yeast at the beginning of the stationary phase. In this system, the overexpression of each form of α-synuclein was highly toxic in the CLS assay, with only about 20% of cells expressing either protein remaining alive by day 3 of the stationary phase, compared to about 70% of cells alive in the empty vector control cells. There was no significant difference seen in cell death between the expression of wild-type or A53T α-synuclein. Deletion of three proteins of interest involved in the apoptotic pathway was found to have no effect on either wild-type or A53T α-synuclein toxicity in the CLS assay. Finally, the authors expressed wild-type or A53T α-synuclein in yeast cells lacking mitochondrial DNA in the CLS assay. Surprisingly, ablation of mitochondrial DNA eliminated the toxic effects of α-synuclein overexpression in yeast cells in the stationary phase, indicating that the α-synuclein toxicity seen in the CLS assay is dependent on functional mitochondria (Buttner et al., 2008).

In another study, Sampaio-Marques et al. (2012) explored α-synuclein-induced toxicity in the CLS assay using a Tet-On system to induce wild-type α-synuclein expression during exponential growth, the diauxic shift, or the stationary phase of the CLS, and then continued following the yeast life span. Overexpression of α-synuclein was found to be toxic when induced at all stages of growth, but CLS, evaluated through CFU, was shortened most significantly when α-synuclein was induced starting in the stationary phase. These results indicate that aged cells may be particularly vulnerable to high levels of α-synuclein. Interestingly, when α-synuclein was induced at day 0 of the stationary phase, post-exponential phase and diauxic shift, and autophagy was inhibited using the pharmacological drug chloroquine, yeast CLS was greatly extended past that of cells overexpressing α-synuclein alone. However, a chloroquine-only treatment group without α-synuclein expression was not included, making this result difficult to interpret, as autophagy may play a significant role in the survival of wild-type cells in the CLS assay as well. As found by Buttner et al. (2008), when expressed from a high-overexpression galactose-inducible system, both wild-type or A53T α-synuclein expression were highly toxic to cells in stationary growth, reducing the number of viable cells to only 10% of the initial amount after 30 h of expression, compared to 100% remaining viable in cells with empty vector or overexpressing an α-synuclein A30P mutant protein (Sampaio-Marques et al., 2012). Under moderate expression throughout cell life, controlled by a constitutive *TPI1* promoter, expression of all three forms of α-synuclein showed toxicity compared to vector control cells, with A53T α-synuclein shortening CLS the most, and A30P moderately reducing yeast CLS. Unexpectedly, when the three forms of α-synuclein were expressed in cells lacking *Atg11*, which encodes a protein involved in autophagy coordination, under the *TPI1* constitutive promoter, only the A30P mutant, thought to be the less toxic form of α-synuclein from previous experiments, was found to have a shortened CLS. No toxicity was seen through overexpression of any form of α-synuclein in a mitophagy-deficient *atg32* deletion strain, indicating that mitophagy is specifically essential for α-synuclein induced CLS shortening (Sampaio-Marques et al., 2012).

Two more recent studies have explored exogenous modulators on α-synuclein toxicity. First, a small study by Lee et al. (2011), presented a screen of the Prestwick and NIH chemical libraries for drugs that protect *S. cerevisiae* from sugar-induced cell death (SICD). SICD is triggered when stationary-phase yeast cells are transferred from spent rich medium into water with 2% glucose and no other nutrients, a paradigm that induces the generation of large amounts of ROS. The authors found that triclabendazole, a drug commonly used to treat liver fluke infections in cattle and humans, and approved for veterinary use in the United States, partially protects against SICD. Further tests found that treatment from culture initiation with triclabendazole, also suppresses the toxicity induced by constitutively expressed α-synuclein. Treatment with triclabendazole increased the average CLS, measured by CFU, of cells overexpressing α-synuclein to 194% of the CLS of cells overexpressing α-synuclein with DMSO control (Lee et al., 2011). Triclabendazole is believed to be effective against liver fluke infections by inhibiting β-tubulin. However, triclabenzadole was found to extend the life span of wild-type yeast cells *over* a DMSO control at concentrations that did not affect microtubule morphology (Lee et al., 2011), consistently with the fact that two drugs known to target microtubules in yeast, benomyl and nocodazole did not extend yeast life span (Lee et al., 2011). Because triclabenzadole effectively protected stationary-phase yeast cells from hydrogen peroxide-induced toxicity, the authors concluded that its protection of yeast cells from α-synuclein-induced cell could involve oxidative stress attenuation (Lee et al., 2011).

Most recently, a study by Guedes et al. (2017), reported that glucose restriction to 0.5% extends CLS in yeast overexpressing wild-type α-synuclein from the constitutive *TPI1* promoter. A similar effect was observed upon deletion of *Tor1*, the target of rapamycin, and both interventions were associated with decreased autophagy, which was maintained at homeostatic levels. Maintenance of autophagy in α-synuclein-expressing cells under CR or upon *tor1* deletion was proposed to be achieved by decreasing levels and activity of the yeast sirtuin Sir2 (Guedes et al., 2017).

#### Synphilin-1

In addition to α-synuclein, additional proteins that have been implicated in PD have been studied using the CLS assay. One of these proteins is Synphilin-1, a known interactor of α-synuclein that was first found using a yeast two-hybrid screen.

Interestingly, while studying this protein, Buttner et al. (2010) observed that whereas overexpression of α-synuclein is much more toxic to yeast than synphilin-1 when overexpressed during exponential growth (dividing cells), expression of synphilin-1 or expression of α-synuclein through the constitutive *TPI1* promoter shortened CLS to a similar extent. Furthermore, co-expression of synphilin-1 along with α-synuclein showed induced higher toxicity to yeast in the stationary phase, shortening their CLS more that expression of either of the proteins alone, thus highlighting the relevance of CLS studies to model neurodegeneration. When synphilin-1 and α-synuclein were expressed in a yeast strain lacking *sir2*, a gene implicated in the process of yeast replicative aging, the toxicity and CLS-shortening induced by expression of the proteins individually or in combination was decreased significantly, although toxicity was still higher when synphilin-1 and α-synuclein were expressed together.

#### Human DJ-1 Protein

It is also known as PD protein 7 and a member of the DJ-1 superfamily of proteins. DJ-1 has been previously shown to inhibit the aggregation of α-synuclein (Shendelman et al., 2004) and mutations in human DJ-1 have been implicated in a form of autosomal recessive early-onset parkinsonism (Bonifati et al., 2003). DJ-1 plays some role in protection from oxidative stress, but how it functions is still unclear. Since yeast contains homologs of proteins belonging to the DJ-1 superfamily, Miller-Fleming et al. (2014) studied how deleting these proteins in yeast would affect cell viability during the CLS assay. In wild-type yeast cells, without the expression of α-synuclein, single deletion of multiple DJ-1 family member homologs in yeast shortened CLS (Miller-Fleming et al., 2014). However, the synergistic effect that deletion of these DJ-1 family members may have on α-synuclein toxicity was not studied in this model.

#### Mutations in *PRKN* or *PARK2*

The coding for the protein Parkin, have been also associated with an autosomal recessive form of juvenile PD (Kitada et al., 1998). Parkin is an E3 ubiquitin ligase, which plays essential roles in mitochondrial quality control and turnover. A yeast model to study the function of the Parkin protein was recently developed, in which wild-type Parkin was constitutively expressed in wild-type yeast cells and grown either in standard glucose media or non-fermentable media containing ethanol and glycerol (Pereira et al., 2015). In standard glucose media, no difference between CLS length was seen between cells with or without Parkin expression. However, when grown in non-fermentable media, cells overexpressing Parkin displayed an extended CLS (Pereira et al., 2015). The effects of Parkin overexpression concomitantly with α-synuclein expression are yet to be studied in the yeast CLS assay.

### Familial Amyotrophic Lateral Sclerosis (ALS)

#### Mutations in the Gene *SOD1*

The coding for the mostly cytosolic form of the Cu–Zn superoxide dismutase, was one of the first genetic causes of familial ALS to be discovered, thus the effects of the deletion and overexpression of the SOD1 protein and SOD1 mutants have been a general research focus and have also been studied using the CLS assay. Across species, mitochondria contain a small fraction of SOD1 in the intermembrane space (Sturtz et al., 2001) and a matrix-located Mn-SOD known as SOD2 (Longo et al., 1996). In the original study by Longo et al. (1996) exploring the role of *SOD1* and *SOD2* in superoxide detoxification and its effect on CLS, single and double deletion yeast were grown in standard conditions and then transferred to sterile water upon entrance into the stationary phase. In these conditions, the Δ*sod2* strain showed slightly shortened CLS compared to wild-type cells, the Δ*sod1* strain showed a moderately shortened CLS, and Δ*sod1*Δ*sod2* yeast displayed a severely reduced CLS. Interestingly, in low aeration conditions in sterile water, the Δ*sod1* strain showed a decreased level of toxicity compared to normoxia, while the Δ*sod2* mutant displayed enhanced toxicity compared to normoxia. The CLS shortening observed from the Δ*sod1*Δ*sod2* strain remained the most extreme. Deletion of *COQ3*, a required gene in the synthesis of coenzyme Q, eliminated the respiratory capacity of the yeast cells, and appeared protective against the deletions of *sod1* and *sod2*, however, the cells quickly died during the diauxic shift of the CLS assay, as respiration is required for entrance into the stationary phase (Longo et al., 1996).

As mentioned earlier, although SOD1 is synthesized in the cytosol, a ∼5% fraction of SOD1 and its copper chaperone Ccs1 are transported into the mitochondria to mature within the mitochondrial intermembrane space (Sturtz et al., 2001). Overexpression of the copper chaperone for SOD1, *CCS*, was found to increase the mitochondrial localization of the SOD1 protein, and notably, yeast cells in which mitochondrial SOD1 was enriched had extended CLS compared to yeast in which SOD1 was expressed without this mitochondrial enrichment (Sturtz et al., 2001). These results suggest that minimizing mitochondrial ROS generation is essential to maintain wild-type life span, although because multiple reports have also documented ROS signaling during exponential growth phase that promotes stress resistance that subsequently extends CLS (Pan et al., 2011; Ocampo et al., 2012), a critical ROS homeostasis needs to be maintained for optimal chronological aging.

Brasil et al. (2013) also studied the effects of *SOD1* or *SOD1* mutant overexpression in wild-type yeast cells. This group specifically studied the differences between overexpression of wild-type *SOD1* and the *SOD1* AV4 mutant, the mutation that has been most commonly associated with familial forms of ALS. As glutathione levels within the cell have also been found to be associated with mutant SOD1 toxicity, and overexpression has been previously seen to reduce mutant SOD1 aggregation, the authors also overexpressed both the wild-type and AV4 SOD1 variants in cells lacking the protein that acts as the first step in glutathione synthesis. In the CLS assay, when cells were transferred to sterile water and kept at 37°C during the stationary phase, overexpression of either wild-type or the AV4 SOD1 mutant significantly extended CLS, though the extension by the AV4 mutant was significantly shorter than that of wild-type SOD1. However, when overexpressed in the strain lacking glutathione synthesis, the protective effects of SOD1 overexpression were abolished entirely, and overexpression of the AV4 mutant shortened CLS (Brasil et al., 2013).

#### Senataxin (SETX)

SETX, which plays a vital role in maintaining RNA transcriptome homeostasis, is an RNA helicase implicated in two neurodegenerative disorders. Dominantly inherited mutations were identified in rare juvenile-onset, motor neuron disease pedigrees in a familial form of ALS (ALS4), whereas recessive mutations were found to cause a severe early-onset ataxia with oculomotor apraxia (AOA2) that is the second most common recessive ataxia after Freidreich’s ataxia (Bennett and La Spada, 2015). Senataxin dysfunction has been studied through the CLS assay by targeting its yeast homolog, Sen1. Specifically, a yeast strain missing the N-terminal region of the Sen1 protein was seen to have a severely shortened CLS in standard conditions compared to wild-type cells. This N-terminal region was previously seen to be necessary for protein–protein interactions (Sariki et al., 2016).

#### TAR DNA-Binding Protein 43 (TDP-43)

It is associated with a spectrum of neurodegenerative diseases, including ALS. TDP-43 has been shown to bind both DNA and RNA and have multiple functions in transcriptional repression, pre-mRNA splicing and translational regulation (Sephton et al., 2011). Yeast overexpressing human TDP-43 protein has been used to model dementia and motor neuron disorders, including ALS, in which this protein has been discovered in inclusion bodies. In this yeast model, expression of TDP-43 or TDP-43 mutants was driven by a galactose-inducible promoter on a low-copy-number plasmid, resulting in an intermediate level of expression when induced. In dividing cells, it has been shown that TDP-43 turnover and toxicity depend in part upon the endocytosis pathway (Liu et al., 2017). As seen in ALS patient tissues, TDP-43 inhibits endocytosis, and co-localizes strongly with endocytic proteins. Furthermore, impairing endocytosis increases TDP-43 toxicity, aggregation, and protein levels, whereas enhancing endocytosis reverses these phenotypes (Liu et al., 2017; Leibiger et al., 2018). In non-dividing cells, induction of TDP-43 expression (wild-type or the TDP-43 Q331K variant implicated in ALS) at the beginning of the stationary phase, shortened CLS compared to vector-carrying control cells. Curtailed CLS was similar for both TDP-43 variants by the third day of stationary phase, although TDP-43 Q331K resulted in a much faster decrease in cell viability at early time points. Conversely, when a high-copy-number plasmid was used, induction of wild-type TDP-43 resulted in a high degree of toxicity that quickly reduced cell viability to ∼20% 12 h after induction compared to control cells that remained near 100% viable at this time (Braun et al., 2011). When mitochondrial DNA was depleted, this severe toxic phenotype from wild-type TDP-43 overexpression was partially suppressed (Braun et al., 2011), as previously reported for yeast cells expressing α-synuclein (Buttner et al., 2008). Therefore, mitochondrial respiration has been proposed to play a major role in TDP-43- and α-synuclein-induced cytotoxicities in stationary-phase yeast cells. These results are interesting and would fit with the reported mitochondrial metabolic abnormalities often seen in patients suffering from neurodegenerative disorders. They are also intriguing because although respiratory defects in yeast (even absence of respiration) are less detrimental when produced experimentally only in the stationary phase, once yeast have accumulated nutrient stores during growth and undergone their metabolic remodeling during the diauxic shift, a minimum 40%-residual cell respiration during exponential growth is required for normal CLS (Ocampo et al., 2012).

### Other Neurological Disorders

A handful of other neurological disorders have also been modeled in yeast and studied using the CLS assay.

#### Friedreich Ataxia

Friedreich ataxia is a hereditary autosomal recessive disease that causes severe neurological dysfunction (Rotig et al., 1997). The disease is caused by lowered expression of the human mitochondrial protein frataxin, which is conserved in yeast. In all organisms, frataxin plays an essential role in iron homeostasis and therefore in critical mitochondrial functions such as heme biosynthesis and iron–sulfur cluster assembly (Rotig et al., 1997). Gakh et al. (2006) studied how different mutations in yeast frataxin affected CLS. Their results showed that mutations that resulted in the inability of frataxin to oxidize iron or the inability to mineralize iron, both significantly reduced yeast CLS with similar severity (Gakh et al., 2006). Thus, both these functions seem equally important to preserving cell viability during CLS.

#### Niemann–Pick Type C (NPC)

Loss of function mutations in human *NPC1* cause the rare, but very severe neurodegenerative disorder called Niemann–Pick type C (NPC). Human NPC1 and its yeast homolog Ncr1 are sphingolipid transporters that localize to vacuole membrane and to the ER. The yeast *ncr1* deletion strain has been studied in the CLS assay. Yeast lacking Ncr1 display a premature aging phenotype (cells grown in standard synthetic media at 26°C) and higher sensitivity to oxidative stress associated with mitochondrial dysfunction and accumulation of long-chain bases (Vilaca et al., 2014). Importantly, deletion of the ceramide-activated protein phosphatases *Pkh1, Sit4* and its activator *cdc55* suppressed *ncr1*-deletion phenotypes but downregulation of *de novo* sphingolipid biosynthesis had no protective effect, suggesting that long-chain bases accumulation and shorten CLS may result from an increased turnover of complex sphingolipids (Vilaca et al., 2014, 2018).

#### Cathepsin D-Related Diseases

The loss of human protein cathepsin D has been linked to several neurodegenerative disorders. Cathepsin D is a protease required for efficient lysosomal protein breakdown, calcineurin signaling, and endosomal sorting (Aufschnaiter et al., 2017). The yeast homolog of cathepsin D is the vacuolar aspartyl protease (proteinase A) named Pep4. A *pep4* deletion yeast strain was found to accumulate ROS and to have shorter CLS in standard conditions. Pointing toward a deleterious oxidative stress in the deletion strain, supplementation of the culture media with quercetin, a dietary flavonoid with antioxidant properties, found in a variety of variety of fruits and vegetables, increased CLS in the Δ*pep4* strain although a positive effect was also observed in wild-type yeast (Alugoju et al., 2018).

## Gene Expression Systems and CLS Models

### Inducible Gene Expression Systems: Advantages and Limitations

To date, most inducible yeast models of (gain of function) neurodegenerative diseases have been created by heterologous expression of human genes under the control of the strong *GAL1* promoter, which is activated by galactose and repressed by glucose. Although these models have provided a significant amount of information, the lack of regulation of expression levels, generally resulting in high levels of expression, often leads to acute toxicity. These acute effects can be advantageous when screening for drugs or genetic suppressors of cytotoxicity but are not the ideal system for analyzing metabolic or physiological disturbances leading to cytotoxicity. Additionally, gene expression under the control of a galactose-inducible promoter introduces a metabolic constraint, since gene expression is induced upon transferring the cells to media containing 2% galactose, which is a fermentable carbon source. This system would prevent studies in non-fermentable, respiratory conditions that can be relevant to the study. For example, the use of metabolism-independent inducers would allow for the study of cell toxicities in situations in which the cells are forced to exclusively respire, creating a better model of the highly oxidative neuronal metabolism. Besides, the use of metabolism-independent inducers allows studies in non-dividing post-mitotic cells using the yeast stationary phase model of aging or CLS (Sinclair et al., 1998), the focus of this manuscript. As explained in the previous sections, in this post-mitotic state, energetic dependence on mitochondrial respiration and concomitant ROS production highly resemble the situation in which neuronal cells age.

To create refined inducible yeast models of neurodegenerative disorders, researchers have tested several systems (Table 2). Sampaio-Marques and colleagues used a Tet-On inducible system to study how age traits potentiate the cytotoxic effects of α-synuclein. In these models, α-synuclein expression was induced from a Tet-On promoter in different phases of yeast growth (exponential, diauxic, or stationary) and CLS was then determined (Sampaio-Marques et al., 2012). The Tet-on system is highly regulatable although tend to be leaky. Importantly, even at low concentrations, the inducer of the system, tetracyclines, are known inhibitors of mitochondrial translation and can therefore provoke mitochondrial proteotoxic stress, leading to changes in nuclear gene expression and altered mitochondrial dynamics and function, which will introduce a confounding variable in experimental settings (Moullan et al., 2015). The use of newly developed tetracycline-based systems that are more sensitive could be an alternative to minimize the dose of the antibiotic that needs to be used; however, even if no apparent mitochondrial toxicity is detected, widespread gene expression changes may sensitize cells to the intended tetracycline-controlled loss or gain of function, still confounding the results (Chatzispyrou et al., 2015).

**Table 2:** Inducible gene expression systems used for the construction of yeast models of neurodegeneration.

Promoter	Description/induction	Properties	Reference
*CUP1*pr	*CUP1* codes for metallothionein, a protein that binds copper and mediates resistance to high concentrations of copper and cadmium (Butt et al., 1984). – Induction with Copper (0 to 2 mM CuSO_4_)	– Tight regulation – Weak promoter – Copper may add toxicity, in combination to expression of toxic proteins.	Ocampo and Barrientos, 2008
*GAL1*pr	– Induction by galactose (usually 2%) – Gratuitous induction can be achieved in a Δ*gal1* strain, in which galactose metabolism is deactivated. Cell can be grown in the presence of any carbon source supplemented with low doses of galactose.	– Strong expression – Gratuitous induction is highly regulatable (0.01 and 0.1% galactose are sufficient to induce expression)	Outeiro and Lindquist, 2003; Willingham et al., 2003
Tet-On promoter	Induction by doxycycline (2 μg/ml)	– Highly regulatable – Basal activity may be significant (leaky promoter). – Doxycycline is a potential mitochondrial translation inhibitor.	Sampaio-Marques et al., 2012
GAL4.ER.VP16 transactivator plus *GAL1*pr	Induction by β-estradiol (5–100 nM)	– Highly regulatable – Expression can be achieved in multiple growth conditions. – To prevent toxicity caused by excess VP16, it is necessary to use weak constitutive promoters (e.g., attenuated *TEF1-7*pr) for the expression of the GAL4.ER.VP16 transactivator. – A galactose-independent LexA-ER-VP6 β-estradiol –inducible system can be used as an alternative (Ottoz et al., 2014)	Ocampo and Barrientos, 2008; Ruetenik et al., 2016

*Regulated promoters enable control over the timing and level of gene expression, which is an essential feature to consider for the generation of yeast models of neurodegeneration. They are suitable when expression of genes is desired at a specific stage of cell growth, or to prevent the build-up of cytotoxic effects induced by the protein being expressed. The table lists the inducible expression systems so far used for the generation of yeast models of neurodegeneration, and briefly describe their advantages and drawbacks.*

In our laboratory, we have focused on modeling HD, based on the expression of polyQ domains of normal and pathological length under the control of different promoters, to test for their advantages and drawbacks. We tested the *CUP1* promoter, two different β-estradiol-inducible *GAL1* promoter systems and the *GAL1* promoter in a Δ*gal1* mutant background (Ocampo and Barrientos, 2008, 2012) (Table 2). Evaluation and comparison of the different systems was performed to assess their tightness, regulation of expression levels, self-toxicity, and effects on metabolism. The use of copper is not recommended since it can promote the generation of ROS. Models created using the *GAL1* promoter in cells in which their galactose metabolism is disabled by disrupting the endogenous *GAL1* gene are a good choice, since they are highly regulatable, metabolically gratuitous, and non-toxic. We have opted to more extensively use β-estradiol inducible models, which are strongly regulated and are based on the constitutive expression of a transactivator fusion protein GAL4.ER.VP16, which is formed by a *GAL4* DNA binding domain, a β-estradiol receptor domain and a VP16 (virus protein 16) transcriptional activator that can activate transcription of a gene placed under the control of a galactose-inducible promoter (*GAL1*pr). These models have been further optimized to eliminate VP16 toxicity by using a weak constitutive promoter (an attenuated version of the *TEF1* promoter) for the expression of the GAL4.ER.VP16 transactivator (Ruetenik et al., 2016). In the absence of the β-estradiol hormone, the fusion protein in this system is repressed by the yeast chaperones from the Hsp90 family (Louvion et al., 1993). Upon media supplementation with β-estradiol, the fusion protein is de-repressed, binds to *GAL1*pr through the *GAL4* DNA binding domain and the VP16 recruits the transcriptional machinery to start transcription of the gene of interest. This system has been already used successfully to create models of neurotoxic proteotoxicity expressing polyglutamine domains (Ruetenik et al., 2016) in the context of chronological aging. Using an alternative galactose-independent transactivator could further improve the β-estradiol inducible models. In this line, the recently developed LexA-ER-AD β-estradiol -inducible system, based on a heterologous DNA-binding domain (LexA) and non-toxic activation domains, can be used as an alternative (Ottoz et al., 2014).

### Alternative Culture Models for the Study of Chronological Life Span

In the classical CLS assays, the age-dependent viability of non-dividing cells is estimated in conditions that involved starvation of exogenous nutrients and reliance on storage carbohydrates, glycogen and trehalose (Cao et al., 2016). However, because many terminally differentiated human cell types rarely starve and some, such as neurons, can consume a large amount of energy (Bourre, 2006), it has been considered imperative to develop a yeast model to study CLS, in which nutrients are not limited, but cell division stops and metabolism remains highly active. With this goal in mind, uncoupling metabolism from cell division, Nagarajan et al. (2014) have promoted the study of CLS in immobilized cells using bioreactors, a technology that has been extensively used in brewing and bioethanol production (Verbelen et al., 2006). To model CLS under nutrient-replete conditions, the authors encapsulated cells in a matrix made of calcium alginate to form beads that were packed into bioreactors and fed *ad libitum* (Nagarajan et al., 2014). In these conditions, cells stopped dividing, retained high glycolytic flux but decreased expression of genes in the tricarboxylic acid cycle, stored large amounts of glycogen, had enhanced expression of stress resistance genes and maintained >95% viability over prolonged culture (17 days). Therefore, yeast metabolism in bioreactors is very different from metabolism in batch cultures, where yeast cells depend on respiration to survive the diauxic shift and reach the stationary phase (Ocampo et al., 2012). Respiratory-competent cells grown in batch liquid cultures in the presence of 2% glucose use both fermentation of stored carbohydrates and respiration for survival once they reach the stationary phase (Ocampo et al., 2012). However, when yeast is grown in liquid cultures containing only 0.5% glucose (what is considered as a model of calorie restriction), their stress resistance is enhanced, accumulation of stored carbohydrates is increased and CLS is extended by 3–4 fold (above 1 month) while their requirement of oxidative metabolism for survival is abolished (Ocampo et al., 2012). Immobilized yeast under calorie-unrestricted bioreactor conditions also exhibits long CLS in the absence of respiration, which is precluded by glucose repression and low O_2_ tension (Nagarajan et al., 2014). In conclusion, the pattern of gene expression and the metabolism of immobilized cells grown in bioreactors is similar to that of stationary phase liquid cultures initially grown in the presence of low glucose concentrations. Despite the value of immobilized yeast cultures, the classical liquid cultures may be easier to implement in most research laboratories and offer the possibility to modulate media composition to allow aging cells to use fermentative and respiratory metabolisms.

## Concluding Remarks

Yeast models of neurodegenerative disorders have traditionally used rapidly dividing cells and expression of human disease genes. Although some disease genes are conserved along evolution, some of those responsible for age-associated neurodegenerative disorders are restricted to vertebrates. While many features of the disease cannot be modeled in yeast (e.g., neuron type specificity or loss of function activities), yeast models have proven to provide a robust paradigm for the elucidation of pathways leading to cell death upon deletion or overexpression of conserved genes, or upon heterologous expression of human disease genes. The refinement of yeast models by the incorporation of neurotoxic gene expression controlled by metabolism-independent promoters has allowed the study of neurotoxic proteotoxicity in non-dividing yeast using the CLS model of aging. Although relatively few studies using the yeast CLS model are found so far in the literature, they are expected to exponentially increase as they can be a source of information regarding the chronology of physiological events leading to neurotoxic proteotoxicity-induced cell death and the identification of new pathways involved. Importantly, the yeast CLS model has been instrumental for the identification of pathways that modulate life span in yeast and higher organisms. Therefore, inducible yeast models of neurodegeneration in the context of CLS will allow testing whether and how life span modulators (e.g., nutrient-sensing and stress-resistance pathways) postpone the physiological effects and cell death induced by neurotoxic proteins.

## Author Contributions

All authors listed have made a substantial, direct and intellectual contribution to the work, and approved it for publication.

## Conflict of Interest Statement

The authors declare that the research was conducted in the absence of any commercial or financial relationships that could be construed as a potential conflict of interest.
